# Assessment of *Aspergillus fumigatus* in Guinea Pig Bronchoalveolar Lavages and Pulmonary Tissue by Culture and Realtime Polymerase Chain Reaction Studies

**DOI:** 10.3390/ijms13010726

**Published:** 2012-01-11

**Authors:** Dennis G. Hooper, Vincent E. Bolton, John S. Sutton, Frederick T. Guilford, David C. Straus, Laura K. Najvar, Nathan P. Wiederhold, William R. Kirkpatrick, Thomas F. Patterson

**Affiliations:** 1 RealTime Laboratories, Inc, 4100 Fairway Court, #600, Carrollton, TX 75010, USA; 2 S&S BioConsulting, LLC, Austin, TX 78660, USA; 3 Your Energy Systems, 5050 El Camino Real, #110, Los Altos, CA 94022, USA; 4 Department of Microbiology and Immunology, Texas Tech University Health Sciences Center, Lubbock, TX 79430, USA; 5 Department of Medicine, The University of Texas Health Science Center at San Antonio, San Antonio, TX 78229, USA; 6 South Texas Veterans Health Care System, San Antonio, TX 78229, USA; 7 Division of Pharmacotherapy, University of Texas at Austin College of Pharmacy, Austin, TX 78712, USA

**Keywords:** *Aspergillus fumigatus*, Realtime PCR, bronchoalveolar lavages

## Abstract

In this study we pursued a diagnostic target in *Aspergillus fumigatus (AF)* by using qualitative Realtime PCR combined with proprietary DNA primers and a hydrolysis probe specific for this fungal target. Qualitative Realtime PCR is a diagnostic tool that utilizes Realtime PCR technology and detects the presence or absence target specific DNA within a predetermined detection range. Respiratory tissue and fluids from experimentally infected guinea pigs were tested by extracting DNA from the samples which were amplified and detected using *AF* specific DNA primers and probe. This study included qualitative evaluations of all specimens for the presence of the DNA of *AF*. The findings in the tissues after *AF* infection were compared to the numbers of spores in aerosolized samples used to inoculate the animals. Results demonstrated that the specific probe and primer set could detect the presence or absence of *AF* DNA in the sample. The qualitative detection limit of the assay ranged from 6 × 10^4^ copies to 6 copies. Since blood cultures are rarely positive for Aspergillosis, our data indicate that qualitative Realtime PCR, in combination with the appropriate DNA primers and probe can serve as an effective diagnostic tool in the early detection of fungal infections.

## 1. Introduction

Invasive fungal infections are the most common infections observed in immunocompromised patients [1]. The incidence of invasive aspergillosis (IA) and other fungal infections has increased significantly over recent decades because of the increasing numbers of surviving immunocompromised patients [2]. Fungal infections in adults and children have been extensively studied; however, the speed necessary to slow these infections and identify the causative organism has fallen short in the effort to reduce the mortality in these patients [3]. The incidence of infectious aspergillosis has increased dramatically over the past two decades in bone marrow transplant recipients [4], yet the ability to diagnose these infections has not increased substantially in laboratory medicine. In the United States alone, greater than 27,000 solid organ transplant procedures were performed in 2008 [5]. Pappas *et al*., in 2010 [6], reported a slight detectable increase in invasive fungal infections (IFIs) during a surveillance period of solid organ transplant patients in the U.S. during the period of 2002–2005. They further reported that a large number of IFIs occurred later than 1 year after transplantation, with as many as 25% of IFIs developing later than 3 years after transplantation. They suggested that these data indicated that prevention, diagnostic and treatment strategies need to be further developed to take these late developing infections into account.

At present, patients suspected of having a fungal infection are subjected to multiple blood, urine, tissue, and other body fluid cultures. Fluid specimens are placed in blood culture bottles and on various selective media and cultured at room temperature and 37 °C for various periods of time. The shortest period of time that various automated microbiological methods can detect fungal infections is 8–12 h, with the longest period of time in automated methods and/or culture media methods being as long as 2–4 weeks. Culture techniques such as these also lack sensitivity. Patterson [7] provided a framework for evaluating genomic targets in animal models to improve the diagnosis and treatment of invasive aspergillosis. This framework evaluated an enzyme linked immunosorbent assay (ELISA) technique that allows for the detection of galactomannan from *Aspergillus fumigatus* (*AF)* [7,8] and incorporates other biomarkers, such as PCR-based diagnosis platforms. As Patterson pointed out, the galactomannan detection in serum and other body fluids, especially bronchoalveolar lavage (BAL) fluid, plays a major role at present in non-culture based diagnosis of IA [7]. However, the sensitivity and specificity of the galactomannan assay is low and fraught with many false negatives and false positives [8,9].

Methods utilizing biological molecular techniques, such as polymerase chain reaction (PCR) or various types of nucleic acid blots, which take time and have serious contamination problems, are currently available and used in many laboratories for this type of testing. However, Realtime Polymerase Chain Reaction (Realtime PCR) offers a clean, closed system which is not prone to contamination observed with other techniques. Realtime PCR instruments such as the Cepheid SmartCycler^®^ and the Applied Biosystems^TM^ 7500 Fast were used for these studies and provided an instrument platform capable of determining the presence or absence of *AF* DNA and virtually eliminates contamination issues prevalent in open system instruments [10–12]. This paper describes work conducted using qualitative Realtime PCR which is a diagnostic tool that utilizes Realtime PCR technology and detects the presence or absence target specific DNA within a predetermined detection range. Qualitative detection assays have been previously described and are currently in use in clinical laboratories worldwide [13–15].

Realtime PCR based methods can be used to amplify, detect and identify specific fungal DNA by utilization of target specific primers and probes. Realtime PCR, using hydrolysis probes [16–17], combines amplification and simultaneous probe hybridization to achieve sensitive and specific detection of infectious fungi (e.g., molds) in Realtime, thereby providing rapid detection of opportunistic fungal pathogens such as *AF*, *A. flavus*, *A. niger* and *A. terreus*. Diagnostic tools available to a clinical lab in the mycology arena are extremely limited. Furthermore, successful treatment outcomes of fungal infections are predicated on effective, early, and sensitive/specific laboratory tests [7]. In the assay described here, primers and a probe were designed to amplify and detect a 136 base pair region on the 18S Ribosomal RNA gene of *AF*. The assay was optimized and validated as directed by the Clinical and Laboratory Institute (CLSI) [18–20]. Efficiency, accuracy, precision, analytical sensitivity (detection limit), and analytical specificity (interfering substances) were the performance characteristics determined in this test with the amplification efficiency of the assay being within the acceptable range of 90 to 105% and the linear standard curve (R^2^) greater than 0.980. Furthermore, the test was audited and approved for human diagnostic purposes by the Centers for Medicare and Medicaid Services (CMS) and the College of American Pathologists (CAP). After inspections of the laboratory, validations and procedures were found to be compliant with Clinical Laboratory Improvement Amendments (CLIA), the test was approved for human molecular diagnostic testing.

In this study, we examined one such promising and untapped diagnostic target in *AF* by using qualitative Realtime PCR combined with the proprietary DNA primers and a hydrolysis probe specific for this fungal target. Respiratory tissue and fluids from experimentally infected guinea pigs were tested by extracting DNA from the samples and amplified and detected using the *AF* specific DNA primers and probe described above.

This study provides data comparing the number of spores found in tissues after infection, with the numbers of conidia in aerosolized samples used to inoculate the animals by semi quantitative culture. In addition, this study includes qualitative evaluations of all specimens for the presence of the DNA of *AF* in the lung tissue and BALs of the tested animals.

## 2. Results and Discussion

**Figure 1:**
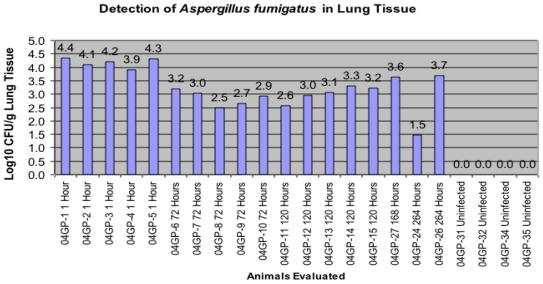
Comparison of infected groups to numbers of *Aspergillus fumigatus* colony forming units (CFU) by culture present in the lung.

Histopathology of the lungs of the guinea pigs demonstrated similar findings as reported by Vallor *et al*. [21] in which conidia were found in the pulmonary alveolar spaces within 1 hour. post infection. As noted in that study, guinea pig lungs were visibly infected with *Aspergillus* lesions compared to lungs obtained from uninfected animals. At one hour post infection, the mean pulmonary fungal burden was assessed by semi-quantitative culture and revealed in this study to be a count of log_10_ 4.23 +/− 0.7 CFU/g). There was a statistically significant decrease in the fungal burden demonstrated at day 3 through day 7, when compared to the 1 hour time point (Figure 1).

All data in Figure 1 are presented as the number of *AF* CFUs by culture and were detected (Log_10_/g Lung) upon sacrifice of the animal and removal of the lung tissue. This evaluation was conducted with each animal infected and sacrificed one hour later through animals being sacrificed 264 hours post-inoculation. Four uninfected animals were also used in this evaluation.

**Figure 2:**
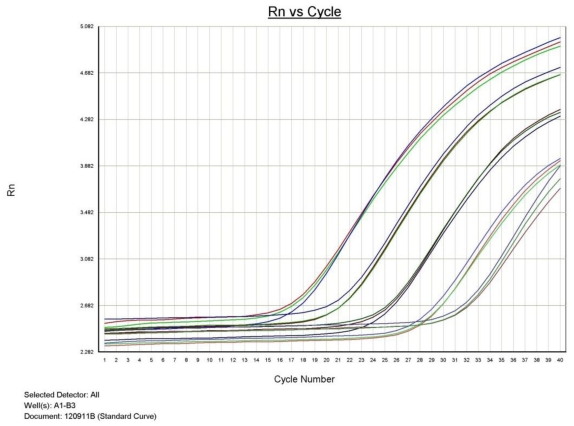
*Aspergillus fumigatus* PCR dilution series demonstrating the qualitative detection limit.

**Figure 3:**
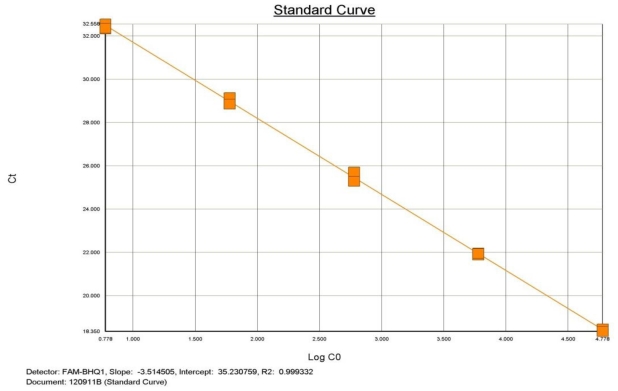
*Aspergillus fumigatus* PCR dilution Standard Curve demonstrating efficiency and linearity.

In addition, this study also demonstrated that qualitative Realtime PCR is capable of detecting very small amounts of target specific DNA (Figure 2 and Figure 3). The curves shown, demonstrate the amplification efficiency of the assay being within the acceptable range of 90 to 105% and the linear standard curve (R^2^) greater than 0.980. The AF assay limit of detection dilution series below shows the detection limit of the assay to be between approximately 6 × 10^4^ copies to 6 copies of purchased AF genomic DNA (See explanation in Materials and Methods)

Approximately 2.0 μg of *AF* DNA was acquired from the American Type Culture Collection (ATCC #1022 D-2). The approximate copy number was determined based on information provided by ATCC and the genome size of *AF*. The template was used in a five point dilution series ranging from 6 × 10^4^ copies to 6 copies. All points of the serial dilution were detected by the ABI 7500 Fast with results demonstrated in Table 1.

Utilizing the qualitative Realtime PCR assay above, the guinea pig bronchoalveolar lavages and lung tissue were evaluated for the presence of *AF* DNA. Results of these studies demonstrated that *AF* DNA could be detected in BALs and lung tissue within the detection limits described above (Table 2).

Conidia were detected by histopathology in the pulmonary alveolar spaces of the guinea pigs within 1 hour post infection, confirming the results of Vallor *et al.* [21]. Also, as seen in the previous study [21], by day 5 post infection, guinea pig lungs showed *Aspergillus* lesions when comparing them to lungs of non-infected animals. These same findings were noted in this study, thus demonstrating the reproducibility of the described animal model. Furthermore, this study determined that when an animal is infected with *AF*, the same organism can be detected by qualitative Realtime PCR in the BALs and lungs. This information could be extremely useful and important in determining the efficacy of many antifungals which may be used in patients who have fungal positive BALs. These studies may be used further to calculate the number of copies to determine prolonging survival when determining efficacy of drugs used in patients who have undergone transplantation or those immunocompromised patients who may succumb to infection by what was initially very few organisms. The results of this study may be expanded to observe the presence or absence of mycotoxins produced by *Aspergillus* sp. (not just *AF*), and to determine the number of copies of fungi needed to actually produce a significant, detectable amount of mycotoxin in a clinical specimen.

## 3. Experimental Section

### 3.1. Animals and Immunosuppression

The animal model assessment of *AF* burden in guinea pigs has been previously described [21]. Briefly, 8 male Harley guinea pigs (0.5 kg; Charles River Laboratories, Wilmington, MA) were made neutropenic with intraperitoneal cyclophosphamide (250 mg/kg; Cytoxan; Mead Johnson, Princeton, NJ, USA) and further immunosuppressed with cortisone acetate (250 mg/kg; Sigma, St Louis, MO, USA) given subcutaneously 2 days prior to inhalational challenge In addition, ceftazidime (100 mg/kg by subcutaneous injections; Glaxo SmithKline Beecham Pharmaceuticals, Philadelphia, PA, USA) was administered daily throughout the study for prevention of bacterial infections. In order to maintain immunosuppression throughout the study, additional doses of cyclophosphamide (200 mg/kg) and cortisone acetate (250 mg/kg) were administered on day 3 post inhalational challenge. All animal research procedures were approved by the Institutional Animal Care and Use Committee at the University of Texas Health Science Center at San Antonio, and animals were maintained in accordance with the American Association for Accreditation of Laboratory Animal Care.

### 3.2. Preparation of Inoculate and Inhalational Challenge

The preparation of *AF* (AF293) has been previously described [21] as has the inhalational challenge [22–24]. Briefly, the organism was cultured; conidia were dislodged from potato dextrose plates, spun in Sorvall centrifuge and, filtered, by approved protocol [21]. A portable, acrylic chamber was used to establish the model of invasive pulmonary aspergillosis (IPA) [23], and was used in this study. The acrylic chamber was used to simultaneously infect groups of guinea pigs with nebulized inocula of 10^8^ CFU/mL of *AF* conidia (total volume was 12 mL). Eighteen animals were exposed to the aerosol mist for 1 hour. After one additional hour, five guinea pigs were sacrificed to confirm and assess the average conidial delivery of the infected run. Guinea pigs were sacrificed by terminal exsanguinations after being anesthetized with 44 mg/kg ketamine-HCl and 10 mg/kg xylazine. A cohort of four immunosuppressed guinea pigs was left uninfected but underwent the same immunosuppression and placebo therapeutic regimen as the infected cohort. After sacrifice, lungs, liver, kidney, brain, bladder, spleen, and serum were collected from each animal. Saline injections to obtain BALs were performed prior to exsanguinations from all animals. Only lung homogenates and BAL results were reported in this study.

### 3.3. Fungal Burden Assessment

Fungal burdens in the lungs of the 15 guinea pigs were determined by CFU counting using the same methods as previous reported [21]. Guinea pigs were infected with 2.3 × 10^7^ CFU/mL of *AF* by aerosolization as previously described [21].

### 3.4. *AF* Qualitative Realtime PCR Detection Limits

Lyophilized *AF* genomic DNA (strain NRRL 163) was acquired from ATCC to determine the detection limits of the *AF* Realtime PCR assay. The lyophilized genomic DNA (approximately 6.18 × 10^7^ copies) was rehydrated in 100.0 μL of PCR grade water for a final concentration of approximately 6 × 10^5^ copies/μL. This DNA solution was used to setup a dilution series from approximately 6 × 10^4^ copies/μL to 6 copies/μL. Upon completion of the dilution series, 1.0 μL of each dilution series point was used in a Realtime PCR reaction.

### 3.5. Extraction and Qualitative Realtime PCR assessment of AF

To ensure maximum recovery of fungal DNA, lung homogenates and BALs were further homogenized using silica beads and a bead beating technique (Sigma, St. Louis, MO, USA) in order to ensure cellular release of genomic nucleic acid from all of the *AF* conidial and hyphal forms present. One milliliter (1.0 mL) aliquots of lung homogenates or BALs were subjected to sterile bead beating for 1.0 minute (Mini Bead Beater, BioSpec Products, Bartlesville, OK, USA) as previously described [21]. Homogenized samples were processed for DNA extraction using the QIAamp DNA Mini Kit (Qiagen, Valencia, CA, USA) according to the manufacturer’s directions. Purified DNA was obtained for all samples processed with the purified DNA used as templates for qualitative Realtime PCR analysis. Proprietary primers and a probe specific for *AF* plus an internal control assay were designed by RealTime Labs, Inc. and synthesized by Integrated DNA Technologies, Inc. (Coralville, IA, USA). Primers were synthesized at a scale of 25 nm and probes were synthesized at a scale of 100 nm.

### 3.6. Assay Controls and Reaction Details

The test was further controlled by using internal controls of *Geotrichum* sp. (GEO) and known positive controls of *AF* as well as known negative samples. The characteristics of the DNA probe and primers in this study were forward primer size: 20 base pairs (bp); reverse primer size 20 bp; and probe size 26 bp. All probes used for the assays were hydrolysis probes with the reporter fluorescent reporter dye, 6-carboxy-fluorescein (FAM) attached to the 5′ end and the quencher Black Hole Quencher (BHQ-1) attached to the 3′ end. Primers and probes were received lyophilized and re-suspended into 100 μM stocks. These stocks were then mixed into working stocks containing probe and primers at required concentrations. The enzyme and reaction buffers used in the reaction were purchased from Cepheid manufactured specifically for the SmartCycler^®^ system and Applied Biosystems manufactured specifically for the 7500 FAST. The reactions were set up per manufacturer recommendations. An internal control assay was run for each extracted sample to insure the sample extracted generated purified DNA and was present in the reaction and contained no inhibitors. Additionally, a positive control and negative control was run for each assay to insure that each fungal Realtime PCR assay component was amplifying and detecting the target and that there were no problems with contamination. All assays and the internal control had a cycling profile containing an initial hot start followed by 45 denature and anneal cycles. All assays were designed and operated as qualitative detection assays and were able to determine the presence or absence of fungal DNA specific to the assay used in the reaction.

### 3.7. Histological Evaluation

Samples of guinea pig lungs were aseptically removed and fixed in 10% neutral buffered formalin. Paraffin-embedded tissue sections were then sectioned and stained with Grocott-Gomori methenamine-silver stain (GMS) and Hematoxylin-Eosin (H and E) stain (Tissue Techniques, Inc., Dallas, TX, USA) for evaluation of the inflammatory response in the immunosuppressed animals as well as for the presence of the fungal organisms.

### 3.8. BAL and Lung Homogenate Evaluation

Five hundred microliters of BALs and frozen tissue from lungs as well as 500 μL of homogenized lung tissue in saline from the guinea pigs were sent to RTL from the University of Texas Health Science Center at San Antonio on dry ice. Three hundred microliters of BAL and 300 μL of lung homogenate were centrifuged at 13,000 rpm for 10 minutes (AcuSpin, microcentrifuge, Fisher Scientific, Germany). The supernatant was removed and the pellet was re-suspended in 50 μL of normal saline and vortexed for 15 seconds. Fifty microliters of the sediment was placed on a glass microscopic slide and a drop of Lactophenol Cotton Blue was added to the sediment. A cover slip was placed on the sample and observed under low power. Spores were counted using a hemocytometer. Results of the spore count are seen in Figure 1. Hyphal elements were also noted.

## 4. Conclusions

This study shows that guinea pigs infected with AF by use of an aerosol mist had detectable fungal burden in their lungs up to 11 days following infection. It was also determined that *AF* DNA could be detected utilizing qualitative Realtime PCR in both bronchoalveolar lavage and lung tissue up to 11 days following infection. Lastly, the detection limit of the qualitative Realtime PCR assay was shown experimentally.

Realtime PCR is a powerful tool for determining the presence or absence of specific fungal targets in body fluids and tissue. Because blood cultures are rarely positive in Aspergillosis, the technology described here has the capability to detect down to one copy of DNA and has the practical ability to routinely detect down to 10 copies of DNA. This study has shown that specific Realtime PCR DNA probes and primers can be utilized in qualitative studies of body fluids and tissues, specifically BALs, to determine the presence or absence of *AF* DNA within the detection limits of the assay. Realtime PCR in combination with a well-designed target specific assay can and will be useful when evaluating transplant patients or immunocompromised patients for the presence of potentially lethal fungal organisms. This diagnostic ability will lead to more successful transplants accompanied by fewer patient complications and deaths.

## Figures and Tables

**Table 1:** Results by qualitative Realtime PCR assay of an *Aspergillus fumigatus* dilution series ranging from approximately 6 × 10^4^ copies to 6 copies.

Points	Approximate concentration of each Point	Result
**1**	6 × 10^4^ Copies	Detected
**2**	6 × 10^3^ Copies	Detected
**3**	6 × 10^2^ Copies	Detected
**4**	6 × 10 Copies	Detected
**5**	6 Copies	Detected

**Table 2:** Detection of Aspergillus *fumigatus* DNA in lung homogenates and bronchoalveolar lavage (BAL).

Animal Identification	Infection Group	Time Animals Sacrificed After infection	*A. fumigatus DNA* Detected in Lung homogenates	*A. fumigatus DNA Detected* in BAL
04GP-1	Infected	At 1 hour	Positive	Positive
04GP-2	Infected	At 1 hour	Positive	Positive
04GP-3	Infected	At 1 hour	Positive	Positive
04GP-4	Infected	At 1 hour	Positive	Positive
04GP-5	Infected	At 1 hour	Positive	Positive
04GP-6	Infected	At 72 hours	Positive	Positive
04GP-7	Infected	At 72 hours	Positive	Positive
04GP-8	Infected	At 72 hours	Positive	Positive
04GP-9	Infected	At 72 hours	Positive	Positive
04GP-10	Infected	At 72 hours	Positive	Positive
04GP-11	Infected	At 120 hours	Positive	Positive
04GP-12	Infected	At 120 hours	Positive	Positive
04GP-13	Infected	At 120 hours	Positive	Positive
04GP-14	Infected	At 120 hours	Positive	Positive
04GP-15	Infected	At 120 hours	Positive	Positive
04GP-27	Infected	At 168 hours	Positive	Positive
04GP-24	Infected	At 264 hours	Positive	Positive
04GP-26	Infected	At 264 hours	Positive	Positive
04GP-31	Not infected	At 264 hours	Not detected	Not detected
04GP-32	Not infected	At 264 hours	Not detected	Not detected
04GP-34	Not infected	At 264 hours	Not detected	Not detected
04GP-35	Not infected	At 264 hours	Not detected	Not detected
